# RAM function is dependent on Kapβ2-mediated nuclear entry

**DOI:** 10.1042/BJ20131359

**Published:** 2014-01-10

**Authors:** Thomas Gonatopoulos-Pournatzis, Victoria H. Cowling

**Affiliations:** *MRC Protein Phosphorylation Unit, College of Life Sciences, University of Dundee, Dow Street, Dundee DD1 5EH, U.K.

**Keywords:** capping, karyopherin β2 (Kapβ2), 7-methylguanosine, mRNA cap methylation, RNA guanine-7 methyltransferase (RNMT), RNMT-activating mini-protein (RAM), HA, haemagglutinin, HEK, human embryonic kidney, IF, immunofluorescence, Kapβ2, karyopherin β2, NLS, nuclear localization signal, pol II, polymerase II, RAD, RNMT-activation domain, RAM, RNMT-activating mini-protein, RNGTT, RNA guanylyltransferase and 5′-phosphatase, RNMT, RNA guanine-7 methyltransferase, WT, wild-type

## Abstract

Eukaryotic gene expression is dependent on the modification of the first transcribed nucleotide of pre-mRNA by the addition of the 7-methylguanosine cap. The cap protects transcripts from exonucleases and recruits complexes which mediate transcription elongation, processing and translation initiation. The cap is synthesized by a series of reactions which link 7-methylguanosine to the first transcribed nucleotide via a 5′ to 5′ triphosphate bridge. In mammals, cap synthesis is catalysed by the sequential action of RNGTT (RNA guanylyltransferase and 5′-phosphatase) and RNMT (RNA guanine-7 methyltransferase), enzymes recruited to RNA pol II (polymerase II) during the early stages of transcription. We recently discovered that the mammalian cap methyltransferase is a heterodimer consisting of RNMT and the RNMT-activating subunit RAM (RNMT-activating mini-protein). RAM activates and stabilizes RNMT and thus is critical for cellular cap methylation and cell viability. In the present study we report that RNMT interacts with the N-terminal 45 amino acids of RAM, a domain necessary and sufficient for maximal RNMT activation. In contrast, smaller components of this RAM domain are sufficient to stabilize RNMT. RAM functions in the nucleus and we report that nuclear import of RAM is dependent on PY nuclear localization signals and Kapβ2 (karyopherin β2) nuclear transport protein.

## Abstract 2

7-Methylguanosine cap formation in mammals is catalysed by RNGTT and RNMT-RAM. RAM activates the cap methyltransferase, RNMT. We define the domains of RAM required for RNMT activation and demonstrate that nuclear localization is dependent on Kapβ2 interaction with RAM PY-NLSs.

## INTRODUCTION

In eukaryotes, RNA pol II (polymerase II) transcripts are synthesized as precursors which undergo a complex series of processing events prior to translation. The first processing event is the addition of the cap, an inverted 7-methylguanosine group joined to the first transcribed nucleotide via a 5′ to 5′ triphosphate bridge [1–4]. In higher eukaryotes the first transcribed nucleotides are also methylated in a variety of species-specific configurations. The cap is uniquely found on RNA pol II transcripts and is critical for transcript expression. The cap structure protects transcripts from exonucleases and recruits complexes, including CBC (cap-binding complex) and eIF4F (eukaryotic initiation factor 4F) complex, which mediate transcription elongation, splicing, nuclear export and translation initiation [1]. The cap is present on the transcript throughout its lifetime and the process of decapping initiates RNA degradation [5].

Nascent transcripts are synthesized with a 5′ triphosphate on the first transcribed nucleotide to which 7-methylguanosine is added by three enzymic activities [1–4]. A triphosphatase removes the terminal phosphate and a guanylyltransferase adds guanosine monophosphate to create the structure G(5′)ppp(5′)X (X is the first transcribed nucleotide). Subsequently, an RNA cap methyltransferase methylates the guanosine cap on the N-7 position to create the basic cap structure, m7G(5′)ppp(5′)X. The capping enzymes are recruited to the phosphorylated RNA pol II C-terminal domain at the initial stages of transcription, which places them in the proximity of the emergent nascent transcript [6,7]. In mammals, the guanylyltransferase and triphosphatase are contained on a single peptide, RNGTT (RNA guanylytransferase and 5′-phosphatase), and the cap methyltransferase is a distinct protein, RNMT (RNA guanine-7 methyltransferase). RNGTT and RNMT are recruited to elongating RNA pol II [8,9], and are rate-limiting for gene expression and cell proliferation [8,10,11].

RNMT activity and expression were found to be dependent on a previously uncharacterized nuclear protein called RAM (RNMT-activating mini-protein) [12]. RAM increases RNMT cap methyltransferase activity 5-fold *in vitro* and in cells RAM is required for transcript cap methylation. RAM also protects the RNMT protein from degradation, although the mechanism involved is not known [12]. Since RNMT expression and function is dependent on RAM, it is perhaps unsurprising that RAM was found to be critical for gene expression and cell proliferation.

**Figure 1:**
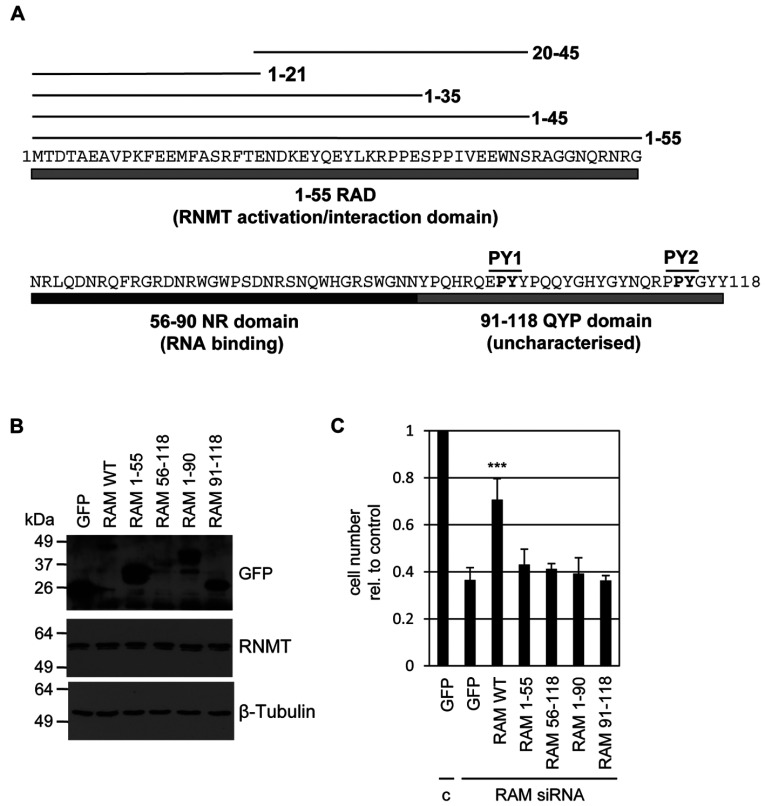
The RAM RAD, NR and QYP domains are required for RAM function (**A**) Depiction of human RAM domains investigated in this study. RAD, amino acids 1–55, interacts with RNMT and stimulates its activity. NR domain, amino acids 56–90, is an RNA-binding domain. QYP domain, amino acids 91–118, is uncharacterized. Two PY NLSs at amino acids 98 and 114 are indicated. (**B**) HeLa cells were transfected with pcDNA5 RAM-GFP, RAM-GFP mutants or GFP alone. At 3 days following transfection, Western blots were performed to detect the antigens indicated in cell extracts. Molecular masses are indicated in kDa. (**C**) HeLa cells were co-transfected with RAM or control siRNA (c), and pcDNA5 RAM-GFP, RAM-GFP mutants or GFP alone. At 3 days after transfection the number of cells relative to siRNA control/pcDNA5 GFP transfection were calculated. The histogram depicts the average for three independent experiments and the error bars indicate±S.D. ***P<0.001 using Student's *t* test in comparison with RAM siRNA/pcDNA5 GFP transfection.

RAM is a 118-amino-acid protein in humans and is conserved in vertebrate species. The amino acid sequence of RAM bears little homology to other human proteins (Figure 1A) [12]. Several functional domains have been characterized (summarized in Figure 1A). The N-terminal 55 amino acids of RAM contain the RAD (RNMT-activation domain), which interacts with RNMT and stimulates cap methyltransferase activity. Amino acids 56–90 form the NR domain, which has an enrichment of arginine and asparagine residues and binds to RNA. The NR domain is not required to increase cap methyltransferase activity *in vitro*; however, it may increase the recruitment of transcripts to RNMT in cells. Amino acids 91–118 form the QYP domain which has an enrichment of tyrosine, glutamine and proline residues, although its function is unknown.

In the present study we determine that all three domains of RAM are critical for RAM function and characterize the QYP domain as a mediator of nuclear import.

## MATERIALS AND METHODS

### Cell culture and transfections

HeLa and HEK (human embryonic kidney)-293 cells were cultured in DMEM (Dulbecco's modified Eagle's medium)/10% FBS, in 5% CO_2_ at 37°C. HeLa cells were transiently transfected with pcDNA5- or pcDNA4-based constructs in 6-well dishes using Lipofectamine™ 2000 (Invitrogen). HEK-293 cells were transiently transfected with pcDNA4-based constructs using calcium phosphate. siRNA was purchased from the Dharmacon siGENOME collection [RAM, D-021286-01; RNMT, D-019525-01; Kapβ2-1 (karyopherin β2-1), D-011308-01; Kapβ2-2, D-011308-02], and transfected using Lipofectamine™ RNAiMAX (Invitrogen). Non-targeting siRNA (D-001210-02) was used as a negative control. Cells were lysed 48 h post-transfection.

### Cell proliferation

HeLa cells (10^5^) were transfected with 50 μM siRNA and 0.5 μg of pcDNA5 HA-RNMT and 0.5 μg of pcDNA5 Fg-RAM or RAM-GFP expression constructs, using Lipofectamine™ 2000 (Invitrogen). The cDNAs utilized were resistant to siRNA via silent mutation of wobble codons. Two days following transfection, cells were counted using a Countess cell counter (Invitrogen).

### Cloning

Constructs were created using standard cloning procedures. Constructs were made resistant to siRNA by site-directed mutagenesis of the siRNA-binding site using the QuikChange® Site-Directed Mutagenesis kit (Stratagene). RAM siRNA 1-resistant cDNA was made using the oligonucleotide 5′-GTT-TGAAGAGATGTTTGCGTCGCGCTTTACGGAGAATGACA-AGGAGTATCAGGAATACCTGAAACG-3′. RNMT siRNA 1-resistant cDNA was made using the oligonucleotide, 5′-AGCC-ATATCCTGCAAATGAGTCCAGCAAGTTAGTCAGCGAGA-AGGTGGATGACTATGAACATGCAGC-3′. All constructs were sequence verified. Primers are available on request.

### Cell extract preparation and immunoprecipitation

Cell extracts were lysed in Triton lysis buffer (10 mM Tris/HCl, pH 7.5, 50 mM NaCl, 50 mM NaF, 30 mM Na_4_P_2_O_7_, 10% glycerol, 0.5% Triton X-100 and protease inhibitors) and debris was removed by centrifugation at 15000 ***g*** for 15 min at 4°C. Protein concentration was determined using the Bradford reagent and diluted to 1 mg/ml. For immunoprecipitations, 0.5 mg of cellular proteins were incubated with 1 μg of polyclonal RNMT antibodies or isotype control plus 25 μl of Protein A/G–Sepharose (Santa Cruz Biotechnology), 10 μl of anti-HA (haemagglutinin) agarose (Sigma) or 10 μl of GFP-Trap (ChromoTek) for 4 h at 4°C. Resins were washed in Triton lysis buffer (10 mM Tris/HCl, pH 7.5, 50 mM NaCl, 50 mM NaF, 30 mM Na_4_P_2_O_7_, 10% glycerol and 0.5% Triton X-100) and resuspended in 50 μl of Laemmli buffer. A total of 20% of the immunoprecipitate and 10 μg of input were resolved by SDS/PAGE.

### Western blotting

Proteins resolved by SDS/PAGE were transferred on to a PVDF membrane (Millipore). Membranes were incubated with polyclonal anti-RNMT antibodies (Cowling laboratory), polyclonal anti-RAM antibodies (Cowling laboratory), monoclonal anti-HA antibodies (Sigma), monoclonal anti-GFP antibodies (Roche) and polyclonal anti-β-tubulin antibodies (Santa Cruz Biotechnology) for 1 h at room temperature (18–22°C). Secondary anti-sheep, anti-mouse and anti-rabbit antibodies, and Pico chemiluminescence reagents (Thermo Scientific) were used according to the manufacturer's instructions.

### Recombinant protein production

pGEX-6P-1-based vectors were transduced into BL21(DE3) *Escherichia coli* cells and grown in LB broth. At a density of *D*_600_=0.6, 1 litre cultures were induced with 0.5 mM IPTG for 16 h at 4°C. Cells were resuspended in 15 ml of lysis buffer (50 mM Tris/HCl, pH 7.5, 150 mM NaCl, 1% Triton X-100, 1 mM EDTA, 1 mM EGTA, 0.1% 2-mercaptoethanol, 0.2 mM PMSF and 1 mM benzamidine) and sonicated 8 times on ice for 15 s. Insoluble material was removed by centrifugation for 30 min at 60000 ***g***. Glutathione–Sepharose (1 ml, GE Healthcare) was incubated with the soluble material for 1 h, washed in lysis buffer and protein was eluted in 5 ml of elution buffer (50 mM Tris/HCl, pH 7.5, 150 mM NaCl, 1 mM EGTA, 0.07% 2-mercaptoethanol, 1 mM benzamidine, 0.03% Brij-35 and 50 mM glutathione). The presence of RAM in the samples was verified by MS.

### *In vitro* GST-fusion purification

Recombinant GST–RAM proteins (0.5 nmol) and 0.1 nmol of recombinant RNMT in 500 μl of Triton lysis buffer were incubated with 15 μl of glutathione–Sepharose at 4°C for 3 h. Glutathione–Sepharose was washed in Triton lysis buffer and resuspended in 50 μl of Laemmli buffer. Purified proteins and inputs were resolved by SDS/PAGE followed by Coomassie Blue staining or Western blotting. The binding assays of RAM and Kapβ2 were performed as described previously [12a]. Briefly, 0.5 nmol of GST–RAM were incubated with 0.2 nmol of Kapβ2 in TB buffer (20 mM Hepes, pH 7.3, 10 mM potassium acetate, 2 mM magnesium acetate, 2 mM EGTA, 2 mM DTT and 20% glycerol). The complexes were purified with glutathione–Sepharose, extensively washed in TB buffer and visualized by Western blotting.

### Cap methylation activity assay

The cap methylation activity assay was performed according to [11]. A guanosine-capped unmethylated substrate ^32^P-labelled on the α-phosphate (Gp*ppG-RNA) was produced as follows. A total of 200 ng of a 55-base strand of *in vitro* transcribed RNA was incubated in a 10 μl reaction at 37°C for 30 min with 100 ng of recombinant human RNGTT, 2 μl (10 μCi) of [α-^32^P]GTP and 1 μl of RNAsin (Promega) in reaction buffer (0.05 M Tris/HCl, pH 8.0, 6 mM KCl and 1.25 mM MgCl_2_). RNA was purified by ammonium acetate precipitation. In the cap methyltransferase assay, 15 nM GST–RAM or mutants were pre-incubated with 15 nM RNMT at 4°C for 15 min. A total of 2 ng of capped RNA was incubated with the proteins and 100 nM *S*-adenosylmethionine at 37°C for 10 min in reaction buffer. Following the reaction, RNA was purified, precipitated and resuspended in 4 μl of 50 mM sodium acetate and 0.25 unit of P1 nuclease for 30 min at 37°C. Cap (Gp*ppG) and methyl-cap (m7Gp*ppG) were resolved in 0.4 M ammonium sulphate TLC using polyethyleneimine–cellulose plates. Standards were visualized by UV light to establish correct migration. Labelled spots were visualized and quantified by autoradiography, and percentage conversion of GpppG into m7GppG was calculated.

### RNA extraction and real-time qPCR

RNA was extracted using GeneJET RNA purification kit (Thermo Scientific). PCR was performed using Quanta Biosciences SYBR Green FastMix for iQ. RNA (500 ng) was converted into cDNA using Quanta qScript cDNA Synthesis kit. cDNA (0.2 μl) was used in real-time PCR reactions. The primers used are available on request. PCR products were sequence verified.

### Fluorescence microscopy

HeLa cells expressing RAM–GFP were fixed with 4% paraformaldehyde in PBS for 10 min, washed in TBST (TBS plus 0.1% Triton X-100) for 5 min and permeabilized with 1% Nonidet P40 in TBST. Cells were washed for 10 min in TBST and counterstained with 1 μg/ml DAPI in TBST. Cells were mounted in 2.5% DABCO (1,4-diazadicyclo[2.^2^.^2^]octane) and visualized by fluorescence microscopy (Zeiss LSM 700). Endogenous RAM and RNMT IF (immunofluorescence) analysis was performed as described previously [12]. The nuclear to cytoplasmic ratio of GFP-tagged RAM mutants was calculated using the Volocity software (PerkinElmer). Briefly, DAPI staining was used to define the nuclei and phalloidin and tubulin staining was used to define the whole cell. The cytoplasm was defined by subtracting the nuclei from the whole cell. The average nuclear and cytoplasmic intensity of GFP–RAM mutants from multiple cells in a single image was calculated. The nuclear to cytoplasmic ratio was estimated and the average±S.D. from 12 independent images is presented.

## RESULTS

### The RAD, NR and QYP domains of RAM are required for cell proliferation

In order to characterize and explore the relationship between the different RAM functions, a series of mutants were constructed. Full-length human RAM is 118 amino acids. The truncation mutants created were the RAD, amino acids 1–55; the NR domain (RNA-binding), amino acids 56–90; and the uncharacterized QYP domain, amino acids 91–118 (Figure 1A). Previously RAM had been demonstrated to be rate-limiting for mammalian cell proliferation; however, the critical domains had not been defined [12]. Therefore HeLa cells were depleted for endogenous RAM using RAM siRNA and transfected with pcDNA5-based constructs which express RAM–GFP or GFP alone (Figure 1B). The RAM–GFP constructs used throughout the present study have silent mutations which generate transcripts resistant to RAM siRNA. Two days following transfection, cells were counted and expressed as values relative to cells transfected with control siRNA and GFP alone (Figure 1C). As observed previously, inhibition of RAM expression resulted in a reduction in cell number, in this case to an average of 0.36-fold of the control for three independent experiments. Expression of RAM–GFP partially rescued cell proliferation, resulting in 0.71-fold cells relative to control. Expression of GFP fusions of RAM 1–55, 56–118, 1–90 and 91–118 did not rescue the growth defect of cells depleted of endogenous RAM, with the average cell number not significantly different to that of the GFP control. Therefore the RAD (RAM 1–55), the NR domain (RAM 56–90) and the QYP domain (RAM 91–118) are all critical for RAM function.

### RAM 1–45 is necessary and sufficient to activate RNMT

**Figure 2:**
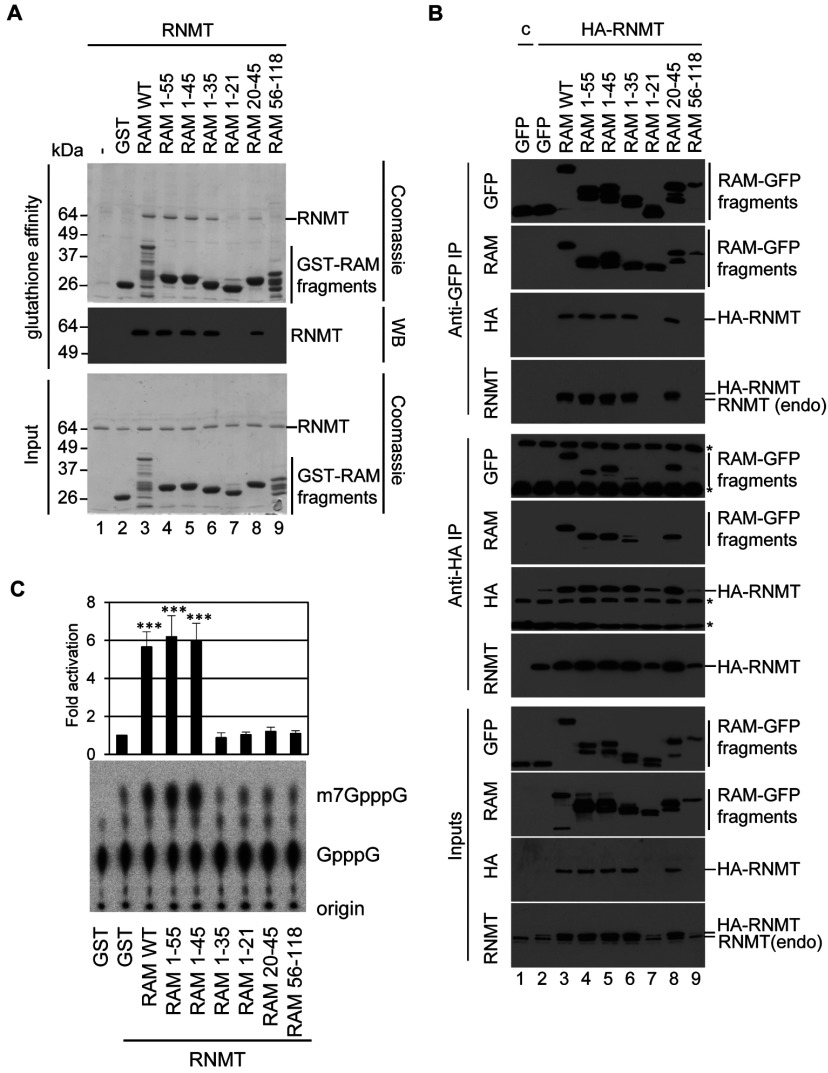
RAM 1–45 activates RNMT (**A**) Recombinant GST–RAM WT, truncation mutants or GST alone were incubated with recombinant RNMT. GST–RAM complexes were purified on glutathione–Sepharose, resolved by SDS/PAGE and co-purified RNMT was visualised by Coomassie Blue staining and Western blotting (WB). Molecular masses are indicated in kDa. (**B**) HeLa cells were transfected with pcDNA5 HA-RNMT or pcDNA5 (c), and pcDNA4 RAM-GFP (RAM WT), RAM-GFP mutants or GFP. Immunoprecipitations (IP) were performed with anti-HA and anti-GFP antibodies. Western blots were performed to detect GFP, RAM, HA and RNMT in inputs and immunoprecipitates. (* indicates cross-reacting antibody heavy or light chain). (**C**) Cap methyltransferase assay was performed using 15 nM RNMT plus 15 nM GST–RAM, truncation mutants or GST control. Protein complexes were incubated with [^32^P]GpppG transcript and *S*-adenosylmethionine for 10 mins. Following the reaction, transcripts were digested and GpppG and m7GpppG were resolved by TLC and visualized by phosphoimaging. A representative image is shown. The average fold change in cap methyltransferase activity compared with that generated by RNMT alone for six independent experiments is depicted. Error bars indicate±S.D. ***P<0.0001 using Student's *t* test for results in comparison with RNMT plus GST. endo, endogenous.

In order to further probe the mechanisms by which RAM activates RNMT, the domains of RAM which interact with RNMT were mapped. The RNMT-interaction domain was previously mapped to the first 55 amino acids of RAM [12]. In order to determine whether smaller fragments of RAM maintain significant interaction with RNMT, a panel of RAM fragments was investigated (Figure 1A). The direct interaction of RNMT and RAM was investigated using recombinant proteins. GST–RAM and mutants were incubated with RNMT and GST–RAM complexes were purified on glutathione–agarose. RNMT co-purifying with GST–RAM and mutants was detected by Coommassie Blue-stained SDS/PAGE and Western blotting (Figure 2A). RNMT was found to bind to GST–RAM, but not GST alone. RNMT also bound to GST–RAM 1–55 and 1–45 equivalently to GST–RAM WT (wild-type), and with lower affinity to GST–RAM 1–35 and 20–45. An interaction between recombinant RNMT and GST–RAM 1–21 or 56–118 was not detected.

The RNMT–RAM interaction was analysed in mammalian cells with similar results. HA–RNMT, GFP–RAM and RAM mutants were expressed in HeLa cells and their interaction was detected by immunoprecipitations performed with anti-GFP and anti-HA antibodies (Figure 2B). In anti-GFP antibody immunoprecipitations, RAM–GFP, but not GFP alone, was observed to interact with HA–RNMT, visualized by Western blotting to detect RNMT and the HA tag (Figure 2B, lanes 2 and 3). In anti-HA immunoprecipitations, HA–RNMT was observed to interact with RAM–GFP, but not GFP alone, visualized by Western blotting to detect RAM and the GFP tag. Anti-HA antibodies did not interact with RAM–GFP when expressed in the absence of HA–RNMT (Supplementary Figure S1 at http://www.biochemj.org/bj/457/bj4570473add.htm). In the anti-GFP immunoprecipitates, an interaction was observed between RAM–GFP 1–55, 1–45, 1–35, 20–45 and HA–RNMT. Conversely, in the anti-HA immunoprecipitates, an interaction was observed between HA–RNMT and the RAM–GFP mutants 1–55, 1–45, 1–35 and 20–45 (Figure 2B, lanes 4–9).

Previously, we demonstrated that the first 55 amino acids of RAM are sufficient to activate RNMT *in vitro* [12]. Since we observed that smaller fragments of this region could interact with RNMT (Figures 2A and 2B), we investigated whether these same RAM fragments are sufficient to activate RNMT. Recombinant GST–RAM was incubated with RNMT and an *in vitro* cap methyltransferase assay was performed. Briefly, RNMT and RAM were incubated with the methyl donor, *S*-adenosylmethionine, and a guanosine-capped transcript, G(5′)ppp(5′)X. Following the reaction, the proportion of G(5′)ppp(5′)X N-7 methylated on the guanosine was quantified (Figure 2C). As observed previously, RAM WT and RAM 1–55 stimulated cap methyltransferase activity more than 5-fold compared with GST control. RAM 1–45 also stimulated RNMT activity equivalently to the WT protein, whereas the smaller RAM fragments, 1–21, 1–35, 20–45 and 56–118, had no effect on RNMT activity. Thus, although the fragments 1–35 and 20–45 can interact with RNMT, they are not sufficient to even partially activate the enzyme.

### RNMT is stabilized by fragments of RAD

**Figure 3:**
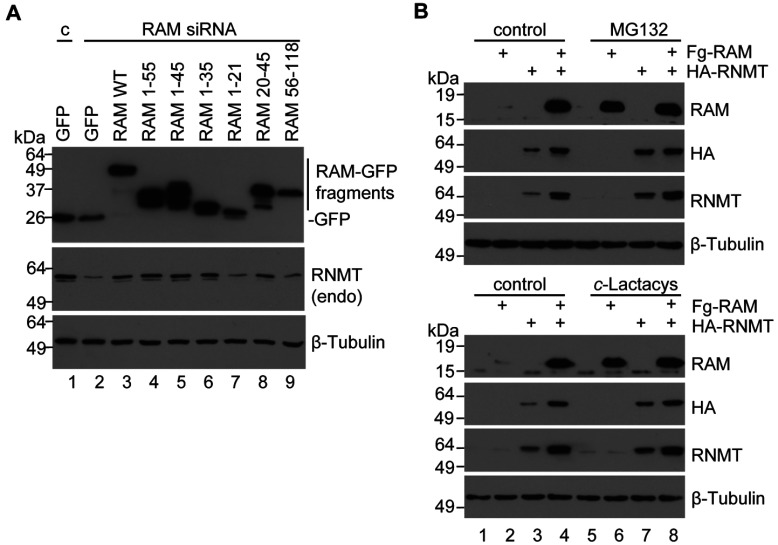
RAM stabilizes RNMT against proteosomal degradation (**A**) HeLa cells were transfected with RAM or control (c) siRNA, and pcDNA4 RAM-GFP or RAM-GFP mutants. Western blots were performed to detect GFP, RAM, RNMT and β-tubulin. (**B**) HeLa cells were transfected with pcDNA5 Fg-RAM and pcDNA5 HA-RNMT alone or in combination with the relevant vector controls. Two days following transfection, cells were incubated with 10 μM MG132, 20 μM clasto-lactacystin β-lactone (*c*-Lactacys) or vehicle control at 8 h before lysis. Western blots were performed to detect RAM, HA, RNMT and β-tubulin in cell extracts. Molecular masses are indicated in kDa. endo, endogenous.

A key function of RAM is to stabilize RNMT protein [12]. The domains of RAM required to stabilize RNMT were investigated using the RAM mutant panel. In Figure 3(A), pcDNA5 RAM-GFP and mutants were transfected into mammalian cells. Endogenous RAM expression was reduced by transfection of RAM siRNA, resulting in reduced RNMT expression (Figure 3A, lanes 1 and 2), and co-transfection of RAM–GFP WT rescued RNMT expression (Figure 3A, lane 3). Endogenous RNMT expression was also rescued by expression of RAM–GFP 1–55, 1–45, 1–35 and 20–45. This confirms that all RAM fragments that interact with RNMT, including RAM 1–35 and 20–45, can rescue RNMT expression.

We investigated whether RAM stabilizes RNMT against proteasome-mediated degradation (Figure 3B). HeLa cells were transiently transfected with pcDNA5 Fg-RAM and pcDNA5 HA-RNMT, individually or in combination. As had been seen previously, transfection of either construct alone resulted in relatively low RAM and RNMT expression (Figure 3B, lanes 2 and 3), whereas co-transfection of both constructs resulted in significantly elevated expression of RAM and RNMT (Figure 3B, lane 4). Incubation of cells with the proteasome inhibitors MG132 or clasto-lactacystin β-lactone for 8 h prior to lysis increased RNMT and RAM expression when cDNAs were transfected individually to levels approaching those found in the RNMT–RAM co-transfection (Figure 3B, lanes 6 and 7). However, when RNMT and RAM were co-expressed, proteasome inhibitors had negligible effect on their expression (Figure 3B, lane 8). These data are consistent with the RNMT–RAM complex protecting both proteins from proteasome-mediated degradation.

### RAM QYP domain contains a nuclear localization motif

RAM is a protein which functions in the nucleus to activate and stabilize RNMT. RNMT has three classical lysine/arginine NLSs (nuclear localization signals), and mutation of all three simultaneously results in mislocalization of RNMT and loss of cell viability [13]. RNMT can be imported independently of RAM, since a mutant of RNMT, which does not bind RAM, is recruited to the nucleus [8]. Conversely, the mechanism by which RAM enters the nucleus is not known, including whether it requires RNMT.

**Figure 4:**
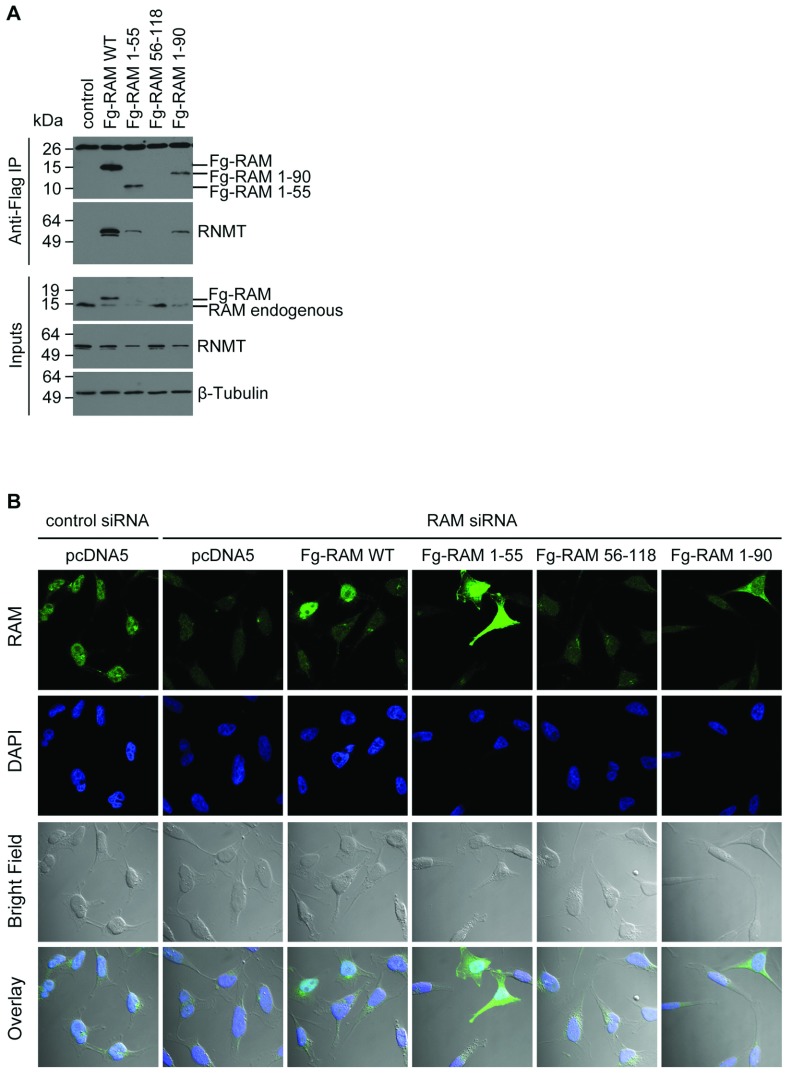
RAM nuclear localization is dependent on the C-terminus (**A**) HeLa cells were transfected with pcDNA5 Fg-RAM WT, truncation mutants or vector control. Immunoprecipitations (IP) were performed on normalized cell extracts using anti-Fg antibody–agarose conjugates. Western blots were performed to detect Fg-tagged proteins, RNMT and β-tubulin in immunoprecipitates and extracts. Molecular masses are indicated in kDa. (**B**) HeLa cells were transfected with control or RAM siRNA and with pcDNA5 Fg-RAM WT, truncation mutants or vector control. IF analysis was used to detect RAM localization and DAPI staining was used to detect nuclei. The overlay of RAM IF, DAPI staining and bright field is also presented.

**Figure 5:**
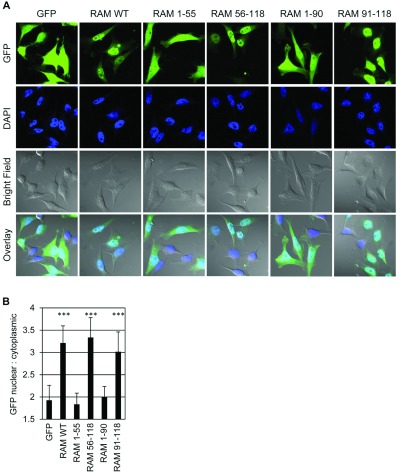
RAM nuclear localization is dependent on the C-terminus (**A**) HeLa cells were transfected with pcDNA5 RAM-GFP, RAM-GFP mutants or GFP. Fluorescence microscopy was used to detect GFP localization in HeLa cells and DAPI staining was used to detect nuclei. An overlay of GFP fluorescence, DAPI staining and bright field is presented. (**B**) The ratio of nuclear to cytoplasmic GFP fluorescence was quantified. The average for 12 images is presented and error bars indicate±S.D. ***P<0.0001 using Student's *t* test for average ratio in comparison with GFP control.

To investigate the RAM domains governing nuclear entry, HeLa cells were simultaneously transfected with RAM siRNA to suppress expression of the endogenous protein and with pcDNA5-based constructs to express Fg-RAM WT and mutants (Figures 1A and 4A). The Fg-RAM cDNAs used throughout the present study have silent mutations which renders them resistant to RAM siRNA. IF performed using polyclonal anti-RAM antibodies was used to detect RAM (Figure 4B). Anti-RAM antibodies were used rather than anti-Fg antibodies, since we have not found the latter to be effective at detecting Fg–RAM by IF (T. Gonapoulos-Pournatzis and V.H. Cowling, unpublished work). The anti-RAM antibodies were raised against the WT protein and recognize epitopes throughout RAM (Figure 2) [12]. In control transfections, endogenous RAM was detected as a nuclear protein by co-localization with the DNA stain DAPI. Transfection of RAM siRNA inhibited the detection of RAM, confirming the specificity of the anti-RAM antibodies. Fg–RAM WT was detected predominantly in the nucleus, whereas Fg–RAM 1–55 and 1–90 were distributed throughout the nucleus and cytoplasm, suggesting that amino acids 91–118 are required for nuclear entry. However, Fg–RAM 1–55 and Fg–RAM 1–90 are poorly expressed, and Fg–RAM 56–118 and 91–118 were undetectable by Western blot or IF (Figure 4A and results not shown). Furthermore, some of these truncation mutants may be small enough to enter the nucleus by passive diffusion and therefore may not provide useful information about the localization of the WT protein. Owing to the limitations of Fg–RAM mutant expression, the subcellular localization of RAM–GFP WT and mutants was also investigated (Figure 5A). The RAM–GFP mutants used, 1–55, 56–118, 1–90 and 91–118, were all detectable by IF (Figure 5A) and Western blotting performed on cell extracts (Figure 1B). GFP expressed alone was distributed throughout the nucleus and cytoplasm, whereas RAM–GFP was predominantly nuclear (Figure 5A). Similar to Fg–RAM, RAM–GFP 1–55 exhibited a localization defect and was distributed throughout the nucleus and cytoplasm, whereas RAM–GFP 56–118 and 91–118 were predominantly nuclear. Since RAM 56–118 and 91–118 do not bind to RNMT (Figure 2), RAM does not require RNMT for nuclear entry. To quantify these observations, the nuclear to cytoplasmic ratio was determined for RAM–GFP and mutants using confocal microscopy (Figure 5B). RAM–GFP WT, 56–118 and 91–118 had a significantly increased nuclear to cytoplasmic ratio when compared with GFP alone. Conversely, RAM–GFP 1–55 and 1–90 exhibited a nuclear to cytoplasmic ratio equivalent to GFP alone and therefore a putative NLS maps to RAM 91–118.

### RAM PY motifs are required for nuclear entry

**Figure 6:**
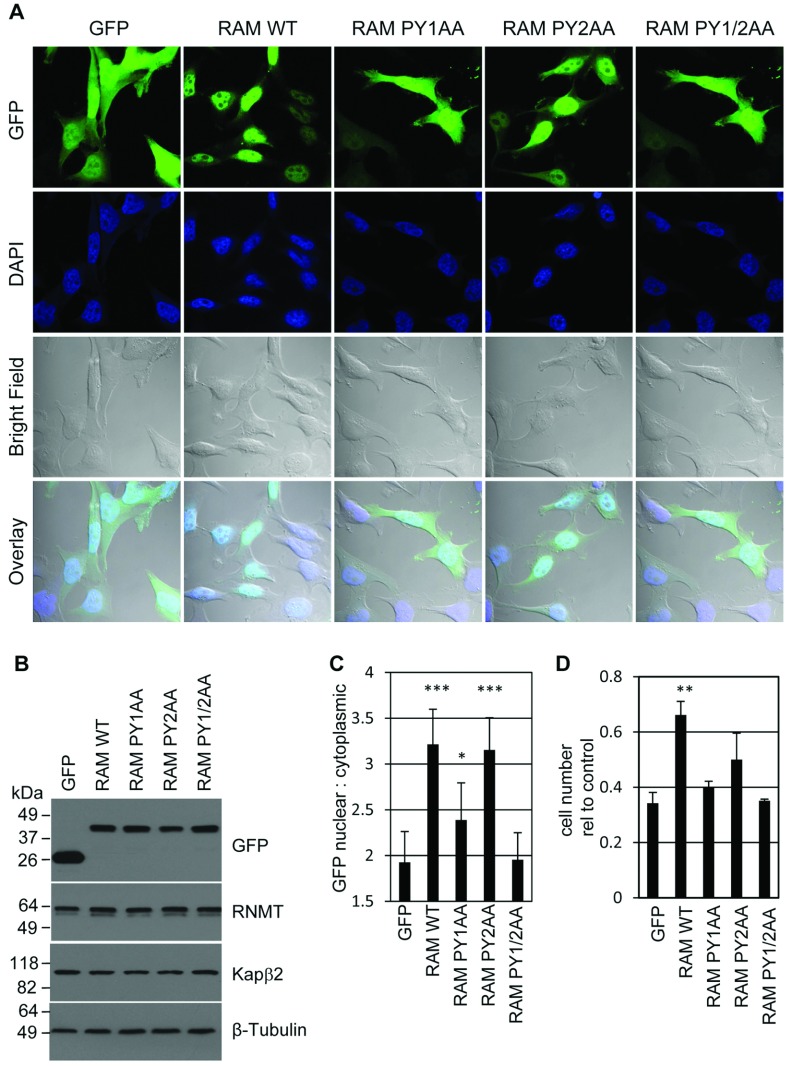
RAM nuclear localization is dependent on the PY domains (**A**) HeLa cells were transfected with pcDNA5 RAM-GFP, RAM-GFP PY/AA mutants or GFP control. Fluorescence microscopy was used to detect GFP localization and DAPI staining was used to detect nuclei. An overlay of GFP fluorescence, DAPI staining and bright field is presented. (**B**) Western blots were performed to detect GFP, RNMT, Kapβ2 and β-tubulin in cell extracts. Molecular masses are indicated in kDa. (**C**) The ratio of nuclear to cytoplasmic GFP fluorescence was quantified. The average of 12 images is presented and error bars indicate±S.D. ***P<0.0001 and *P<0.01 using Student's *t* test for average ratio in comparison with the GFP control. (**D**) HeLa cells were co-transfected with RAM or control siRNA, and pcDNA5 RAM-GFP, RAM-GFP PY/AA mutants or GFP. At 3 days after transfection cells were counted and the number expressed as relative to control/pcDNA5 GFP transfection. The histogram depicts the average for three independent experiments and the error bars indicate±S.D. **P<0.001 using Student's *t* test relative to GFP control.

RAM does not contain a classical lysine/arginine-based NLS, but the QYP domain (91–118) does contain two putative PY-NLSs surrounding P^98^Y (PY1) and P^114^Y (PY2; Figure 1) [14,15]. PY-NLSs mediate nuclear localization via binding to the nuclear transport protein Kapβ2 [16]. Three mutants were created to study the role of the putative RAM PY-NLSs. PY1AA has P98A/Y99A mutations, PY2AA has P114A/Y115A mutations and PY1/2AA has a combination of these mutations. RAM–GFP and PY mutants were expressed in HeLa cells resulting in equivalent expression (Figure 6B). The subcellular localization of these mutants was investigated using confocal microscopy (Figure 6A). RAM–GFP PY1/2AA was mislocalized to the cytoplasm and its average nuclear to cytoplasmic ratio was not significantly different from the GFP control (Figure 6C). In this experimental scenario the most influential NLS is PY1, since the PY1AA mutation had a significant effect on the RAM–GFP nuclear to cytoplasmic ratio, whereas the PY2AA mutation did not. However, since mutating both PY motifs simultaneously had the greatest effect on the nuclear to cytoplasmic ratio, both motifs appear to contribute to localization.

Since the PY mutants are defective for nuclear entry, their ability to support cell proliferation was investigated (Figure 6D). HeLa cells were depleted for endogenous RAM using RAM-directed siRNA and transfected with pcDNA5 RAM-GFP, PY mutants or GFP alone. After 3 days cells were counted and expressed as values relative to cells transfected with the control siRNA/pcDNA5 GFP (Figure 6D). Inhibition of RAM expression resulted in a reduction in cell number, to an average of 0.34-fold compared with the control for three independent experiments. Expression of RAM–GFP partially rescued cell proliferation resulting in 0.66-fold cells relative to control. Cells expressing RAM–GFP PY1AA, PY2AA and PY1/2AA did not proliferate at a significantly different rate to the GFP control.

### Kapβ2 mediates RAM nuclear entry

**Figure 7:**
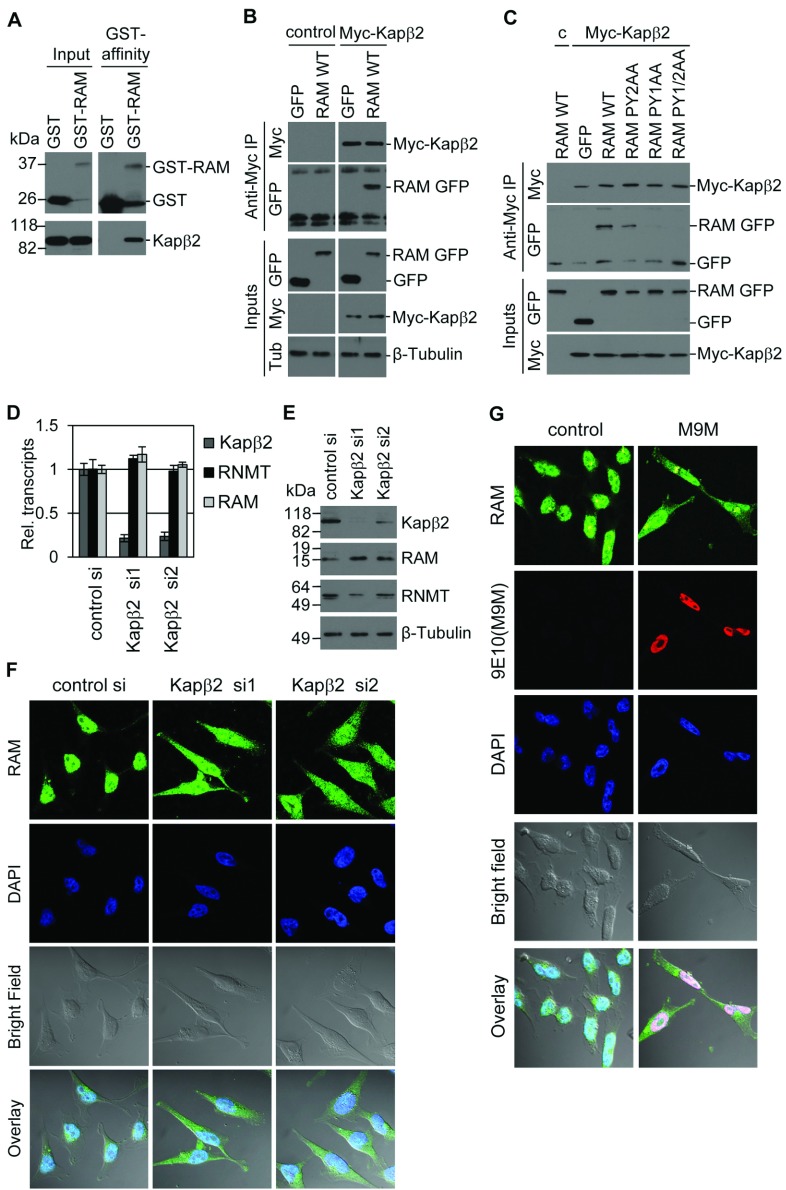
Kapβ2 mediates RAM import (**A**) Recombinant GST–RAM or GST was incubated with recombinant Kapβ2. GST–RAM and Kapβ2 complexes were purified on glutathione–Sepharose, resolved by SDS/PAGE and visualized by Western blotting. Molecular masses are indicated in kDa. (**B**) HeLa cells were transfected with pcDNA5 Myc-Kapβ2 or vector control and pcDNA5 RAM-GFP or GFP. Immunoprecipitations (IP) were performed on cell extracts using anti-Myc antibodies. Western blots were performed to detect Myc–Kapβ2, RAM and β-tubulin in inputs and immunoprecipitates. (**C**) HeLa cells were transfected with pcDNA5 Myc-Kapβ2 or vector control (c) and pcDNA5 RAM-GFP, RAM-GFP PY/AA mutants or GFP. Immunoprecipitations were performed on cell extracts using anti-Myc antibodies. Western blots were performed to detect Myc–Kapβ2, RAM and β-tubulin in inputs and immunoprecipitates. (**D**) HeLa cells were transfected with two independent Kapβ2 siRNAs or control siRNA. After 2 days RNA was extracted and real-time PCR performed to detect expression of Kapβ2, RNMT and RAM. The average result for three independent experiments is presented and the error bars indicate±S.D. (**E**) Western blots were performed to detect Kapβ2, RNMT, RAM and β-tubulin in cell extracts. (**F**) IF was used to detect RAM localization and DAPI staining was used to detect nuclei. The overlay of RAM IF, DAPI staining and bright field is also presented. si, siRNA. (**G**) HeLa cells were transfected with pcDNA3.1 Myc-M9M or vector control. IF was used to detect RAM and Myc-M9M. DAPI staining was used to detect nuclei. The overlay of RAM or RNMT, DAPI staining and bright field is presented.

Since the PY-NLSs are required for nuclear entry of RAM, the functional relationship between RAM and the nuclear transport protein Kapβ2, which binds to PY-NLSs, was investigated [15,16]. To investigate whether RAM and Kapβ2 interact directly, recombinant GST–RAM and GST were incubated with recombinant Kapβ2. GST–RAM and GST were purified by glutathione–agarose and Kapβ2 was only detected in a complex with GST–RAM (Figure 7A). The Kapβ2–RAM interaction was also observed in mammalian cells (Figure 7B). Myc–Kapβ2 and GFP–RAM were expressed in HeLa cells. Immunoprecipitation of Myc–Kapβ2 from cell extracts using anti-Myc tag (9E10) antibodies resulted in co-immunoprecipitation of GFP–RAM, but not GFP alone. The interaction of Kapβ2 with RAM was inhibited by the PY1AA or PY1/2AA mutations and reduced by the PY2AA mutation (Figure 7C). This is consistent with the PY1AA mutation exhibiting a greater defect in nuclear localization compared with the PY2AA mutation (Figure 6C). If the Kapβ2–RAM interaction is mediating nuclear entry, then inhibiting Kapβ2 expression should reduce RAM nuclear localization. Kapβ2 expression was inhibited with two independent siRNAs and a reduction in Kapβ2 transcript and protein was observed (Figures 7D and 7E). A reduction in Kapβ2 expression did not alter RAM expression (Figures 7D and 7E), but did result in RAM mislocalization to the cytoplasm (Figure 7F), indicating that Kapβ2 is rate-limiting for nuclear entry of RAM. A desirable experiment at this stage would be to reverse the observations made with Kapβ2 siRNAs using co-transfection of siRNA-resistant Kapβ2 cDNA. However, we were unable to overexpress Kapβ2, even using inducible constructs (results not shown). As independent evidence that Kapβ2 mediates nuclear entry of RAM, cells were transfected with a plasmid encoding Myc-tagged M9M, a chimaeric PY-NLS peptide which adheres to Kapβ2 effectively blocking cargo binding [17]. M9M was detected by IF performed with 9E10 antibodies, which recognize the Myc epitope. The presence of M9M inhibited nuclear import of RAM consistent with Kapβ2 mediating nuclear import of RAM (Figure 7G).

**Figure 8:**
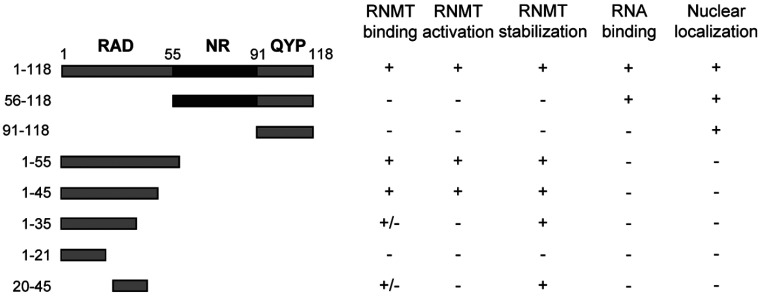
Summary of RAM functional domains Depictions of the functionality of the RAM mutants used in the present study and previously [12].

RNMT localization was not detectably influenced by interference with RAM or Kapβ2 function. Expression of RAM truncation mutants (Supplementary Figure S2A at http://www.biochemj.org/bj/457/bj4570473add.htm), inhibition of Kapβ2 expression (Supplementary Figure S2B) or inhibition of Kapβ2 activity (Supplementary Figure S2C) did not alter RNMT nuclear localization. Thus RAM and RNMT are imported into the nucleus using independent mechanisms (Figure 8). Once nuclear, RAM stabilizes and activates RNMT.

## DISCUSSION

Formation of the 7-methylguanosine cap structure on RNA pol II transcripts mediates key events throughout its lifetime and is critical for gene expression [1]. The enzymes which catalyse its addition, RNGTT and RNMT–RAM, are rate-limiting for gene expression and cell proliferation, and their nuclear localization is required for cell viability [10,12,13]. We previously described three domains of the RNMT activating mini-protein: RAM, an N-terminal RAD; a central RNA-binding domain (NR domain); and a C-terminal uncharacterized domain which we called the QYP domain on the basis of the enrichment of glutamine, tyrosine and proline residues. In the present study we observe that all three domains of RAM are required to support cell proliferation (Figure 1).

### RAM nuclear localization is mediated by Kapβ2

Cap methylation is a process which occurs predominantly during the early stages of transcription, and therefore nuclear entry of RAM is critical for its function of activating and stabilizing RNMT. A major finding of the present study is the identification of the mechanism by which RAM is imported into the nucleus. We demonstrate that RAM has two PY-NLSs based around P^98^Y (PY1) and P^114^Y (PY2), which co-operate to promote nuclear import of RAM. The PY-NLS motif has a loose consensus sequence described as consisting of N-terminal hydrophobic or basic motifs and a C-terminal RX_2–5_PY motif [14]. Since the consensus sequence (and structure) is loose, PY motifs can only be confirmed by experimentation. Mutation analysis demonstrated that simultaneous mutation of both RAM PY-NLSs causes a localization defect indistinguishable from the GFP control (Figure 6). PY1 has a more significant role in nuclear import than PY2, since mutation of PY1 results in a more significant defect than mutation of PY2. PY motifs are recognized by Kapβ2 (importin β), which binds to cargos and nucleoporins, thus targeting cargos to the nuclear pore complex [15]. We established that Kapβ2 binds to RAM directly via an interaction with the PY motifs (Figure 7). Inhibiting Kapβ2 expression with siRNA or inhibiting Kapβ2 function with the M9M inhibitor inhibited RAM import, confirming the functional interaction of Kapβ2 and RAM. Therefore RAM, in common with many other RNA-binding proteins, utilizes Kapβ2-dependent nuclear entry [18–22].

RNMT is imported into the nucleus by an alternative mechanism to RAM. RNMT contains three functional classical nuclear localization signals and binds to the importin-α–importin-β heterodimer [13]. Previously, we demonstrated that RNMT import does not require RAM. RNMT mutants which do not bind to RAM do not exhibit a localization defect [8]. In agreement with this finding, in the present study we confirm that import of endogenous RNMT is not significantly affected when the RAM–Kapβ2 import mechanism is inhibited (Supplementary Figure S2). However, we note that, although we cannot detect a defect in RNMT nuclear localization when we interfere with the RAM–Kapβ2 interaction or function, we cannot discount that a fraction of RNMT is imported in a complex with RAM (and vice versa).

### RNMT activation requires the entire interaction domain

Previously we had established that the first 55 amino acids of RAM are sufficient to activate RNMT. In the present study we determine that, although smaller fractions of this region are sufficient to interact with RNMT and stabilize the protein (Figures 2 and 3), the first 45 amino acids of RAM are necessary and sufficient to activate RNMT (Figure 2C). Currently the effect of RAM 1–45 on RNMT structure is unknown and further studies will be required to determine whether RAM alters the RNMT active site conformation and/or substrate and product affinities.

In summary, we have identified RAM as having three domains; an N-terminal activation domain (RAD), a central RNA-binding domain (NR domain) and a C-terminal nuclear localization domain (QYP), which contains two PY-NLSs (Figure 8). The QYP domain is critical for RAM to enter the nucleus, where it activates RNMT resulting in mRNA cap methylation.

## Online data

Supplementary data
