# Supplementary material for: RAM function is dependent on Kapβ2-mediated nuclear entry
Source: Biochem J. 2014 Jan 10;457(Pt 3):473–84. doi: 10.1042/BJ20131359 (PMC3898117; doi:10.1042/BJ20131359)
Supplement: Supplementary data [file bj4570473add.pdf]

Biochem. J. (2014) **457**, 473–484 (Printed in Great Britain) doi:10.1042/BJ20131359

SUPPLEMENTARY ONLINE DATA

RAM function is dependent on Kapβ2-mediated nuclear entry

Thomas GONATOPOULOS-POURNATZIS*[1] and Victoria H. COWLING*[2]

*MRC Protein Phosphorylation Unit, College of Life Sciences, University of Dundee, Dow Street, Dundee DD1 5EH, U.K.

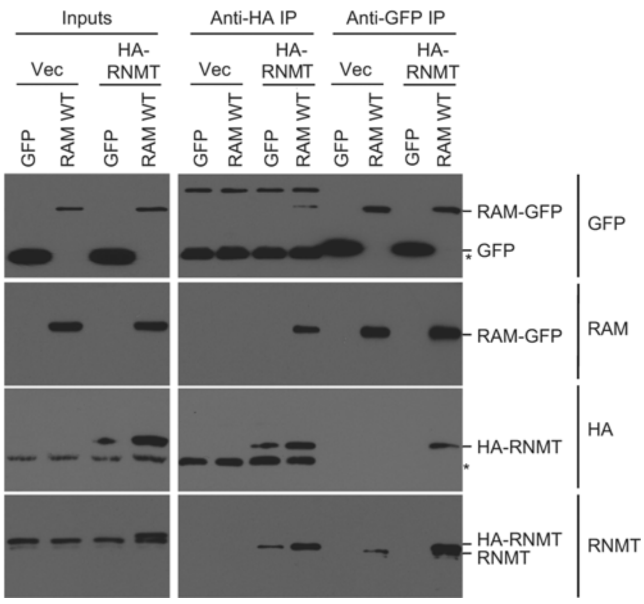

Figure S1 RAM and RNMT interact

HeLa cells were transfected with pcDNA5 HA-RNMT or vector (Vec) control and pcDNA4 RAM-GFP or GFP vector control. Immunoprecipitations (IP) were performed with anti-HA or anti-GFP antibodies. Western blots were performed to detect GFP, RAM, HA and RNMT in the inputs and immunoprecipitates. * indicates cross-reacting antibody heavy or light chain.

[1] Present address: Banting and Best Department of Medical Research and Donnelly Centre, University of Toronto, 160 College Street, Toronto, Ontario, Canada, M5S 3E1

[2] To whom correspondence should be addressed (email v.h.cowling@dundee.ac.uk).

© 2014 The Author(s)

The author(s) has paid for this article to be freely available under the terms of the Creative Commons Attribution Licence (CC-BY) (http://creativecommons.org/licenses/by/3.0/) which permits unrestricted use, distribution and reproduction in any medium, provided the original work is properly cited.

Biochemical Journal www.biochemj.org

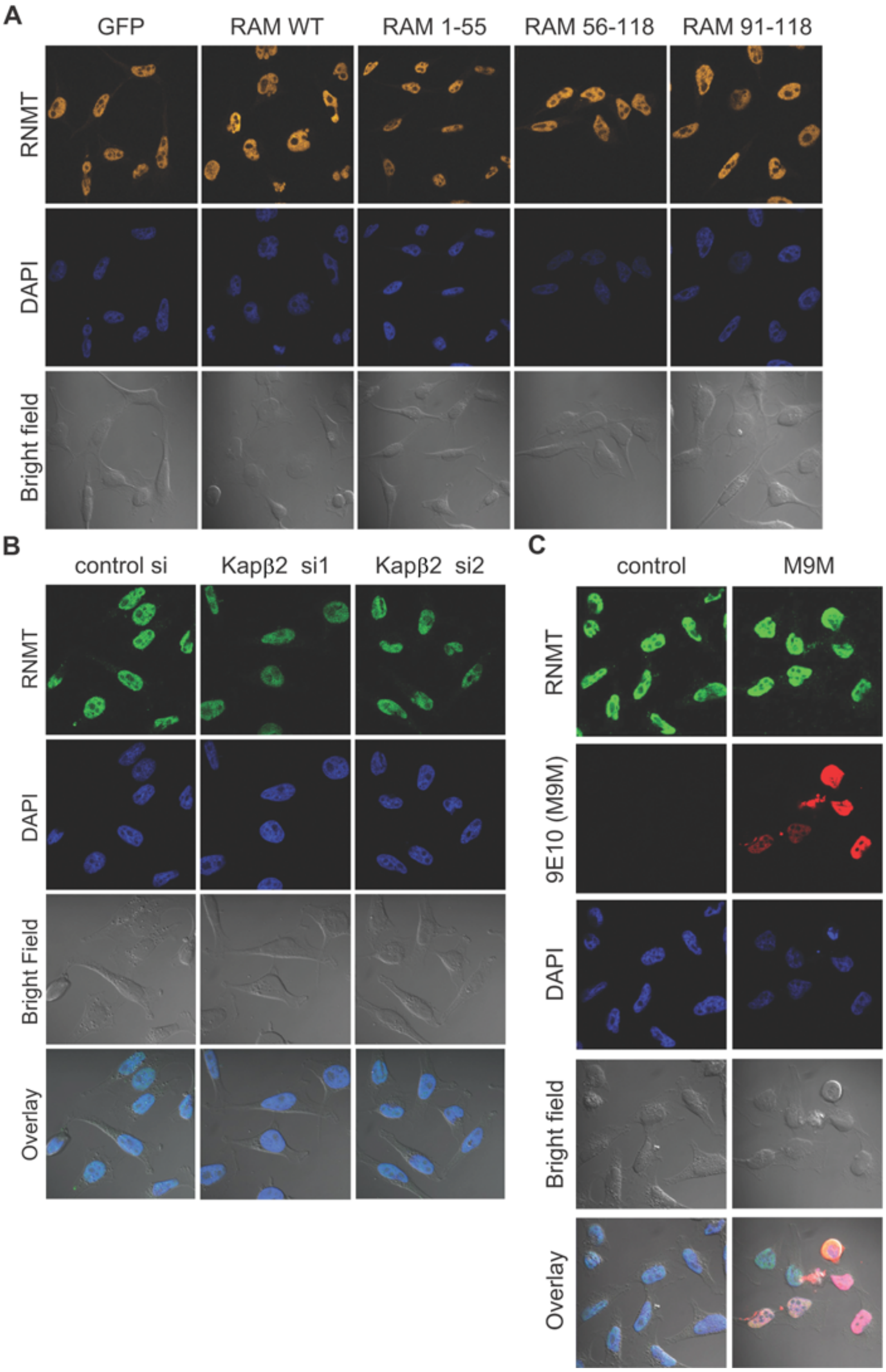

Figure S2 RNMT nuclear localization is independent of RAM

(**A**) HeLa cells were transfected with RAM–GFP WT, truncation mutants (RAM 1–55, RAM 56–118 and RAM 91–118) or GFP vector control. IF microscopy was used to detect RNMT and DAPI staining was used to detect nuclei. (**B**) HeLa cells were transfected with control or two independent Kapβ2 siRNAs (si). IF microscopy was used to detect RNMT localization and DAPI staining was used to detect nuclei. (**C**) HeLa cells were transfected with pcDNA3.1 Myc-M9M or vector control. IF was used to detect RNMT and Myc–M9M. DAPI staining was used to detect nuclei. The overlay of RNMT, DAPI staining and bright field is presented.

Received 14 October 2013/5 November 2013; accepted 7 November 2013
Published as BJ Immediate Publication 7 November 2013, doi:10.1042/BJ20131359

© 2014 The Author(s)
The author(s) has paid for this article to be freely available under the terms of the Creative Commons Attribution Licence (CC-BY) (http://creativecommons.org/licenses/by/3.0/) which permits unrestricted use, distribution and reproduction in any medium, provided the original work is properly cited.
